# The state of ultra risk of psychosis: about a quiz

**DOI:** 10.1192/j.eurpsy.2026.12191

**Published:** 2026-07-31

**Authors:** R. Bendada, I. Semsili, Z. El Maataoui

**Affiliations:** Hôpital Ar-Razi, Salé, Morocco

## Abstract

**Introduction:**

The ultra-high-risk state for psychosis refers to a crucial prodromal phase characterized by early and subthreshold symptoms, such as altered perceptions, ideas of reference, and behavioral changes, which precede the first psychotic episode. This phase presents an opportunity for intervention to delay or prevent progression to full psychosis, particularly in vulnerable individuals.

**Objectives:**

To assess the extent to which psychiatrists consider ultra-high-risk states for psychosis in their clinical evaluations and to analyze care practices adapted to the evolving needs of patients.

**Methods:**

A questionnaire was administered to psychiatrists, covering their practices in assessing ultra-high-risk states, recommended treatments, family support, and therapeutic adjustments based on symptom progression.

**Results:**

The results indicate variability in the integration of ultra-high-risk states for psychosis. Some psychiatrists incorporate prodromal evaluation for a personalized approach, while others prefer standardized treatments. Family support and therapeutic adjustments are seen as essential for comprehensive care.

**Conclusions:**

This study highlights the importance of personalized care based on identifying ultra-high-risk states for psychosis. Integrating the prodromal phase and therapeutic adjustments contributes to optimizing psychiatric care, thereby meeting patients’ specific needs.

**Disclosure of Interest:**

None Declared

